# Co-grinding Effect on Crystalline Zaltoprofen with β-cyclodextrin/Cucurbit[7]uril in Tablet Formulation

**DOI:** 10.1038/srep45984

**Published:** 2017-04-03

**Authors:** Shanshan Li, Xiang Lin, Kailin Xu, Jiawei He, Hongqin Yang, Hui Li

**Affiliations:** 1 College of Chemical Engineering, Sichuan University, Chengdu 610065, PR China

## Abstract

This work aimed to investigate the co-grinding effects of β-cyclodextrin (β-CD) and cucurbit[7]uril (CB[7]) on crystalline zaltoprofen (ZPF) in tablet formulation. Crystalline ZPF was prepared through anti-solvent recrystallization and fully analyzed through single-crystal X-ray diffraction. Co-ground dispersions and mono-ground ZPF were prepared using a ball grinding process. Results revealed that mono-ground ZPF slightly affected the solid state, solubility, and dissolution of crystalline ZPF. Co-ground dispersions exhibited completely amorphous states and elicited a significant reinforcing effect on drug solubility. UV-vis spectroscopy, XRPD, FT-IR, DSC, ssNMR, and molecular docking demonstrated the interactions in the amorphous product. Hardness tests on blank tablets with different β-CD and CB[7] contents suggested the addition of β-CD or CB[7] could enhance the compressibility of the powder mixture. Disintegration tests showed that CB[7] could efficiently shorten the disintegrating time. Dissolution tests indicated that β-CD and CB[7] could accelerate the drug dissolution rate via different mechanisms. Specifically, CB[7] could accelerate the dissolution rate by improving disintegration and β-CD showed a distinct advantage in solubility enhancement. Based on the comparative study on β-CD and CB[7] for tablet formulation combined with co-grinding, we found that CB[7] could be considered a promising drug delivery, which acted as a disintegrant.

Cyclodextrins (CDs) and cucurbit[*n*]urils (CB[*n*]s) are two types of macrocycles commonly used in supramolecular chemistry to improve drug properties, such as solubility, stability, and bioavailability^1^,^2^,^3^,^4^. Natural CDs are cyclic oligosaccharides containing six (α-CD), seven (β-CD), and eight (γ-CD) glucose units by α-(1,4) linkages. CB[*n*]s are generally synthetic pumpkin-like rigid macrocycles comprising *n (n* = 5, 6, 7, 8, 10) glycoluril units doubly-bridged by methylene linkers^5^. More recently, other twisted CB[*n*]s (*n* = 13, 14, 15) were synthesized and separated^6^,^7^. β-CD (Fig. 1a) was firstly found in 1891^8^ and has been studied for over 100 years as pharmaceutical excipient^9^. However, CB[7] (Fig. 1b) was discovered in 2000^10^ and still has wide research prospects. Currently, most reported studies on CB[7] have focused on the inclusion mechanism with drugs at a molecular level^11^,^12^,^13^. Further developments of CB[7]-containing drug dosage forms, such as tablets for oral delivery, have been seldom described. To our best knowledge, reports discussing the dosage forms of CB[*n*]s are limited, except for Wheate *et al*. who have investigated five different dosage forms: CB[6] in oral solid tablet^14^, CB[6] in topical cream^15^, CB[6,7,8]s in eye drop^16^, CB[7] in implantable hydrogel^17^ and CB[6,7]s in nasal insert^18^. Although CB[7] and β-CD exhibit similar cavity size, water solubility, and low toxicity^19^,^20^,^21^, they manifest different inclusion behaviors because of their structural distinctions^22^. Thus, we comparatively investigated their features in solid-state dispersion and tablet formulation.

Poorly soluble zaltoprofen (ZPF, logP = 4.25, p*K*_a_ = 4.21; Fig. 1c) was chosen as a model drug because its commercial formulation is oral tablet and its crystal form is easily obtained. It is a nonsteroidal anti-inflammatory drug (NSAID) that is more potent for the treatment of inflammatory conditions accompanied by pain than other NSAIDs^23^. Numerous methods, including co-evaporation, co-crystallization, freeze-drying, spray-drying, and co-grinding^24^,^25^,^26^, have been widely utilized to prepare drug–excipient dispersions. Among these methods, co-grinding drugs with macrocycles is regarded as the most suitable for scale-up and environmentally friendly industrial production^27^. Nevertheless, when utilizing co-grinding method, there is one thing worthy of being considered. Solely grinding drug without excipient (mono-grinding) may also destroy the drug crystal structure by shear force, even into amorphous forms^28^,^29^, then make more or less effect. Thus, to verify the effects of co-grinding on the drug properties were caused by the adding of macrocycles, the effect of mono-grinding on drug must be excluded.

This study aimed to understand the co-grinding effect of β-CD and CB[7] on the physicochemical properties of crystalline ZPF and to explore their corresponding influence on tablet formulation. Crystalline ZPF was prepared through anti-solvent recrystallization, and its structure was determined by single-crystal X-ray diffraction. Co-ground dispersions (ZPF−CB[7], ZPF−β-CD) were prepared by co-grinding process, and the mono-ground ZPF was also obtained under the same grinding condition as control. Interactions in co-ground dispersions were investigated by using UV-vis spectroscopy, X-ray powder diffraction (XRPD), Fourier transform infrared spectroscopy (FT-IR), differential scanning calorimetry (DSC), solid-state NMR spectroscopy (ssNMR), scanning electron microscopy (SEM) and molecular simulation. The effects of mono-ground ZPF, physical mixtures, and co-ground dispersions on the solubility of crystalline ZPF in water, simulated gastric fluid (SGF), and simulated intestinal fluid (SIF) were also evaluated. Blank tablets with various contents (0%–50%) of β-CD and CB[7] were designed to reveal their specific roles in tablet hardness and disintegration performance. The influence of co-ground dispersions on ZPF tablets was further analyzed through disintegration and dissolution tests. This work provided a basis for the development of new dosage forms by co-grinding method and explored the features of using CB[7] as a tablet excipient in pharmaceutical production.

## Results

### Single-crystal X-ray diffraction

Anti-solvent recrystallization method was utilized to obtain crystalline ZPF. Specifically, crystalline ZPF for X-ray diffraction (Fig. S1A) was obtained through dissolving commercial ZPF in dichloromethane, and *n*-hexane (V/V = 3:7) was gradually added without stirring at room temperature. X-ray diffraction data were collected on an Xcalibur E X-ray single crystal diffractometer and the structure was solved with Olex2^30^, and refined with SHELXS-97^31^ using least square minimization. The determined crystal structure is illustrated in Fig. S1B, and the corresponding crystallographic data are summarized in Table S1 (CCDC No. 1520356). Each ZPF ligand is hydrogen-bonded to an uncoordinated carboxy oxygen atom of an adjacent dimer [O–H···O 2.653(2) Å], resulting in a wavy network that stacks via *π*-*π* interactions into a 3D architecture (Fig. S2). Microcrystalline ZPF (Fig. S1C) used for tablet formulation was prepared by the same solvent system but with vigorous stirring with *n*-hexane addition. The microcrystalline ZPF produced in this manner consists of particles with sizes ranging from 10 μm to 130 μm with a mean of 50 μm.

### UV-vis spectroscopy and molecular docking study

**Figure 2:**
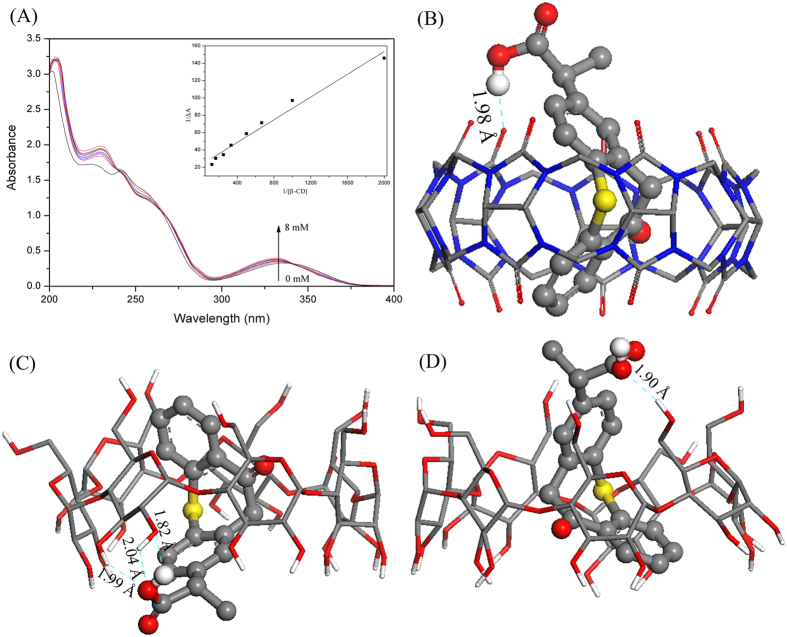
(**A**) Absorption spectra of ZPF in the presence of various concentrations of β-CD from 0, 0.5, 1.0, 1.5, 2.0, 3.0, 4.0, 6.0, 8.0 × 10^−3^ M; Insert: the Benesi–Hildebrand plot of 1/∆A against 1/[β-CD]. (**B**) Docking conformation of CB[7] and ZPF. (**C**,**D**) Two main docking conformations of β-CD and ZPF.

The interactions between ZPF and CB[7] in solution have been determined by UV-vis spectroscopy previously^32^, and the corresponding binding constant that calculated by Benesi–Hildebrand equation was 276 M^−1^. The interactions between ZPF and β-CD in solution were also evaluated by Benesi–Hildebrand equation here. The absorbance intensity of ZPF (Fig. 2A) shows a hyperchromic effect with increasing β-CD concentrations, suggesting the formation of inclusion complex between ZPF and β-CD. From the Benesi–Hildebrand plot, the binding constant of ZPF-β-CD complex was calculated to be 341 M^−1^.

Molecular docking was used to predict the most favorable structures and interaction details of ZPF-CB[7] and ZPF-β-CD complexes. Due to the symmetrical portals of CB[7], there is only one kind of docking conformation of ZPF-CB[7] complex (Fig. 2B). Two main conformations that ZPF molecule entered into the cavity of β-CD from the wide portal (Fig. 2C) and narrow (Fig. 2D) portal respectively were obtained. Regardless of the different structures of macrocycles, the polycyclic moiety of ZPF molecule, including C = O group and phenyl group, was encapsulated both in the hydrophobic cavity of CB[7] and β-CD, and the carboxyl group formed hydrogen bonds with macrocycles. The diverse inclusion conformations and stronger hydrogen bonds between ZPF and β-CD may result in the larger binding constant.

### Characterization of the microcrystalline and mono-ground ZPF, physical mixtures, and binary co-ground dispersions

**Figure 3:**
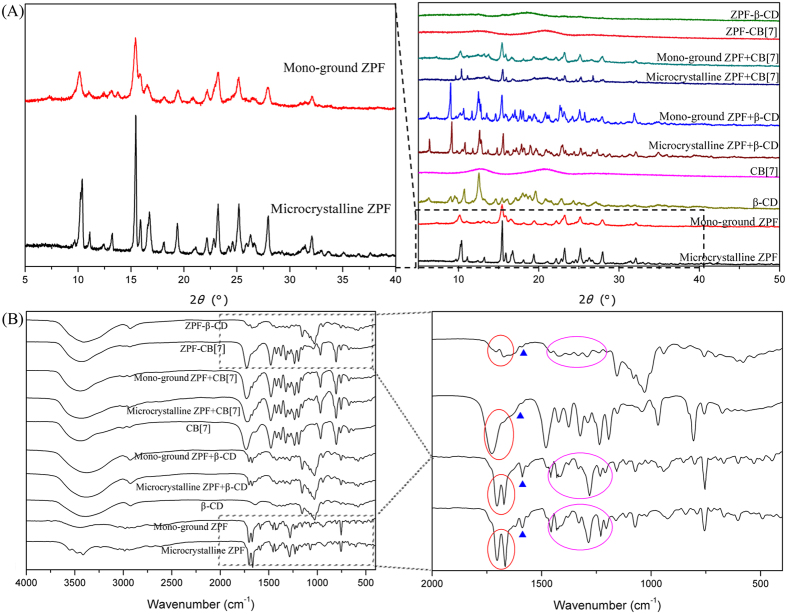
(**A**) XRPD patterns and (**B**) FT-IR spectra of microcrystalline ZPF, mono-ground ZPF and their corresponding physical mixtures and co-ground dispersions (ZPF−CB[7], ZPF−β-CD).

XRPD measurements demonstrated that the characteristic XRPD peaks of microcrystalline ZPF were evidently broadened by grinding solely into mono-ground ZPF for 5 h (Fig. 3A). This finding suggested that the molecularly ordered arrangement of microcrystalline ZPF was partly disrupted and showed a tendency of becoming amorphous. However, ZPF–CB[7] and ZPF–β-CD dispersions exhibited completely amorphous states, indicating that the amorphization of ZPF can be promoted by co-grinding with CB[7] and β-CD. By contrast, the XRPD patterns of physical mixtures presented a simple aggregate of patterns from the corresponding ZPF and macrocycles.

In the FT-IR spectra (Fig. 3B), the characteristic absorption peaks of the mono-ground ZPF were almost the same as that of the microcrystalline ZPF, which presented in the spectra of physical mixtures as well. However, the peaks at 1703 and 1667 cm^−1^, belonging to the stretching vibration of C = O groups, and the peaks at 1588 and 1458 cm^−1^, assigned to the skeleton vibrations of phenyl groups, disappeared after the formation of amorphous dispersions, which demonstrates that the phenyl ring and carbonyl of ZPF interacted with CB[7] and β-CD. This finding is consistent with the result of molecular docking study.

**Figure 4:**
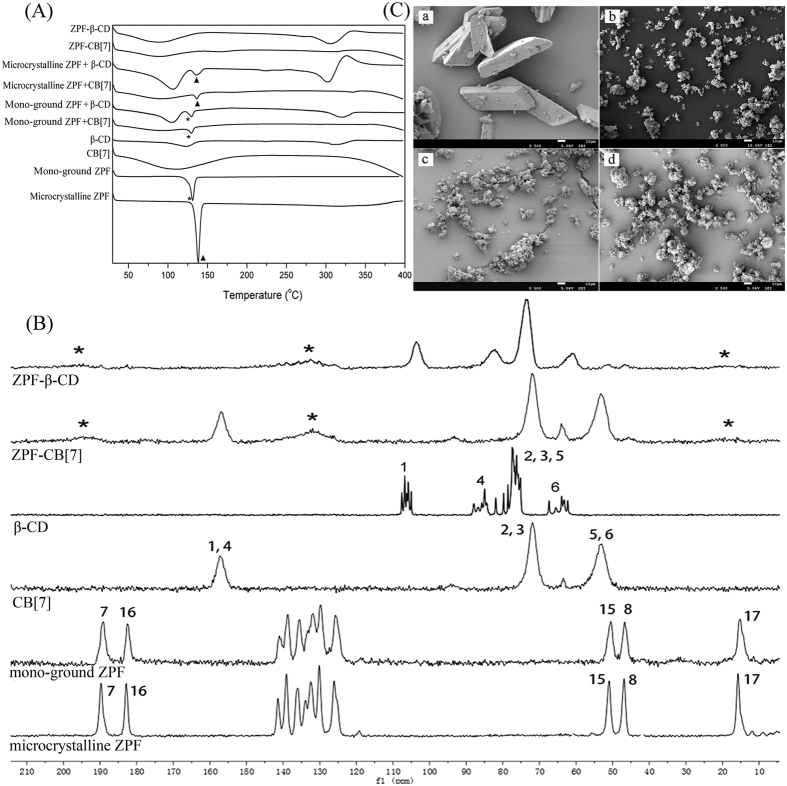
(**A**) DSC curves of microcrystalline ZPF, mono-ground ZPF and their corresponding physical mixtures and co-ground dispersions. (**B**) ^13^C CP/MAS NMR spectra of microcrystalline ZPF, mono-ground ZPF, CB[7], β-CD, ZPF−β-CD and ZPF−CB[7]. Numbering for assignments corresponds to Figure 1. (**C**) SEM images of (a) microcrystalline ZPF, (b) mono-ground ZPF, (c) ZPF−CB[7], and (d) ZPF−β-CD at 500 × magnification.

The DSC curves of microcrystalline ZPF and mono-ground ZPF exhibited a sharp endothermic peak at 138 °C and 131 °C, respectively, which correspond to their melting points (Fig. 4A). When microcrystalline ZPF was ground into mono-ground ZPF by shear force, the melting points varied based on the different solid forms of ZPF. The thermogram of β-CD displayed broad endothermic peaks at approximately 100 °C and 300 °C, which correspond to the release of water molecules and the melting point, respectively. For the DSC curve of CB[7], only a water-released peak was observed because the melting point of CB[7] is beyond 400 °C^33^. The thermograms of physical mixtures ZPF + CB[7] and ZPF + β-CD show the presence of either microcrystalline ZPF or mono-ground ZPF melting peak, indicating that no interaction occurred when the drug and macrocycles were simply mixed. Conversely, the melting peak of ZPF disappeared in both ZPF–CB[7] and ZPF–β-CD amorphous dispersions curves, which indicated that ZPF was embedded by CB[7] and β-CD.

ssNMR provides a direct way to probe the interactions in solid complexes and identify polymorphic forms^34^. Fig. 4B illustrates the ^13^C CP-MAS spectra of microcrystalline ZPF, mono-ground ZPF, CB[7], β-CD, and their corresponding amorphous solid dispersions. The ^13^C CP-MAS spectrum of microcrystalline ZPF exhibited typical crystalline systems with multiple and sharp resonances for each type of carbon. Comparatively broadening signals were observed in the spectrum of mono-ground ZPF, which suggested that the molecular arrangement and intermolecular interactions were partly disrupted after grinding. However, the spectra of ZPF–CB[7] and ZPF–β-CD amorphous dispersions showed the broad featureless resonances of the macrocycle carbon atoms, and the sharp signals of ZPF were distinctly broadened and even merging into the base line after they co-ground with CB[7] and β-CD. This observation demonstrates the loss of overall crystallinity or drug inclusion with macrocycles^35^. Consistent with the XRPD results, this finding confirms their amorphous nature and disordered arrangement and further indicates that solid phase transformation occurs and the dispersions are new solid forms.

The SEM images present the morphology of microcrystalline ZPF, mono-ground ZPF, ZPF–CB[7], and ZPF–β-CD (Fig. 4C). The ZPF crystals showed a prismatic form, whereas the mono-ground ZPF was a granulated aggregation with a much smaller size. ZPF–CB[7] and ZPF–β-CD dispersions also contained smaller particle sizes and exhibited rough, fluffy, irregular surfaces. Furthermore, ZPF co-ground with CB[7] produced more dispersive complexes than β-CD did.

### Solubility studies

The effects of mono-grinding and co-grinding with CB[7] and β-CD on the solubility of microcrystalline ZPF are summarized in Table 1. Grinding ZPF alone for 5 h did not significantly improve the solubility of microcrystalline ZPF. β-CD-containing physical mixtures and co-ground dispersions showed increased ZPF solubility in pure water and SGF. However, no drug solubility improvement was observed for CB[7]-containing physical mixtures in water and SGF. Hence, the solubilization effect of CB[7] is weaker than that of β-CD. CBs are known to preferentially bind guests with positive charges, whereas CDs prefer neutral guest molecules^19^. With neutral ZPF molecules existing in water and SGF, β-CD played a major role in complexation. Interestingly, the co-ground dispersion with CB[7] shows increased ZPF solubility. The solubilization effect of ZPF-CB[7] may be caused by the interactions between CB[7] and ZPF, combining with the disordered molecular arrangement. In SIF, an adverse effect was even observed in the solubility of ZPF by adding β-CD or CB[7]. The different behaviors can be explained by the corresponding ionization equilibria of drugs in the media of different pH values. The ionized and negatively charged ZPF molecules composed of a great proportion in SIF were hard to embed into the hydrophobic cavities of CB[7] or β-CD. A main reason for the decreased solubility in SIF is probably that once a neutral ZPF molecule is complexed with CB[7] and β-CD, the ionization equilibrium shifts and the amount of ionized ZPF molecule is reduced, then the solubility is accordingly reduced. Overall, co-ground dispersions with CB[7] and β-CD were determined to have higher ZPF solubility than its corresponding physical mixtures because of the decreased crystallinity and formed inclusion caused by co-grinding.

### Tablet hardness and disintegration testing

The thickness of blank tablet without CB[7] or β-CD (F0; detailed blank tablet formulation are given in Supplementary Table S2) was set to 2.6 mm to achieve hardness within 4–6 kg. To investigate the effects of β-CD and CB[7] on the disintegrating time of tablet, the hardness must kept consistent (4–6 kg) by adjusting the tablet thickness. The adjusted thickness and corresponding disintegrating time are summarized in Table 2. The tablet thickness was increased gradually as the CB[7] and β-CD contents increased. Therefore, the addition of β-CD or CB[7] instead of lactose enhanced the compressibility of the powder mixture, and the compressibility of CB[7] was better than that of β-CD. Testing the disintegrating time of various β-CD-containing tablets showed that the addition of β-CD almost had no effect on the disintegrating time. Nevertheless, increasing the CB[7] content beyond 15% significantly shortened the disintegrating time. The excellent behavior of CB[7] in disintegration makes it especially suitable as a disintegrant in the preparation of immediate-release tablets.

The disintegrating time of various ZPF tablets (each tablet containing 20% CB[7] or β-CD, and 80 mg ZPF; detailed ZPF tablet formulation are given in Supplementary Table S3) in water, SGF and SIF were evaluated (Table 3). In SGF, each ZPF tablet exhibits the same short disintegrating time. In water and SIF, it is obvious that the addition of CB[7], either in a free state or a co-ground state in tablets showed significant improvement on disintegrating time. Addition of β-CD and mono-grinding ZPF would not make any influence on the disintegration of microcrystalline ZPF tablet.

### ZPF tablet dissolution test

**Figure 5:**
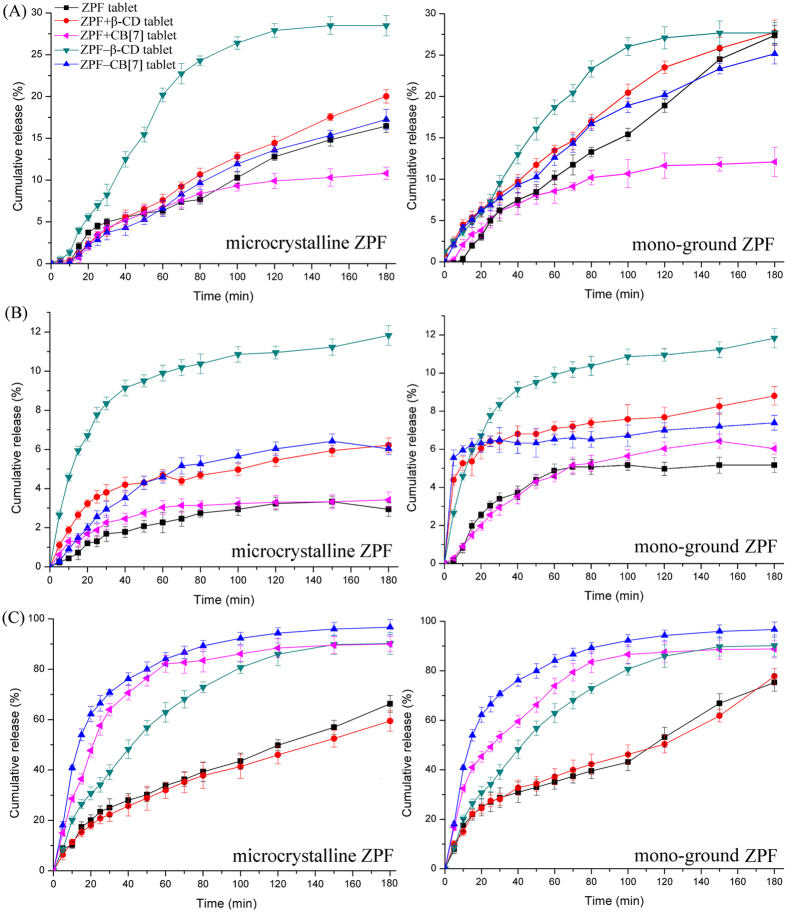
Mean (n = 6) release curves of microcrystalline ZPF, mono-ground ZPF, and their corresponding physical mixtures (ZPF + CB[7], ZPF + β-CD) and co-ground dispersions (ZPF−CB[7], ZPF−β-CD) tablets in (**A**) pure water, (**B**) SGF and (**C**) SIF at 37 ± 0.1 °C under 50 rpm stirring.

The cumulative release curves of microcrystalline ZPF, mono-ground ZPF, and their corresponding physical mixtures (ZPF + CB[7], ZPF + β-CD) and co-ground dispersions (ZPF−CB[7], ZPF−β-CD) tablets in pure water, SGF, and SIF are shown in Fig. 5. Overall, the dissolution rate of microcrystalline ZPF tablet (left in Fig. 5) was not influenced observably by grinding ZPF without macrocycles into mono-ground ZPF (right in Fig. 5). Generally, improving tablet disintegration and solubility of drug would correspondingly increase the drug dissolution rate from tablet. In pure water, CB[7]-containing tablets performed much shorter disintegrating time than that of β-CD-containing tablets (Table 3). But contrary, the dissolution rate of CB[7]-containing tablets were slower than that of β-CD-containing tablets. β-CD-containing tablets exhibited a more significant enhancement in dissolution rate as well in SGF. This finding suggests that although CB[7]-containing tablets disintegrated quickly, the solubilizing ability of β-CD played a major role in increasing the dissolution rate when the dissolution medium is pure water or SGF. Nevertheless, CB[7]-containing tablets showed the fastest dissolution rate in SIF. This drug release performance demonstrated that when the drug solubility was not improved in SIF, the excellent behavior in disintegration of CB[7] accelerated the drug dissolution rate. Thus, β-CD and CB[7] could both accelerate the drug dissolution rate via different mechanisms. Specifically, CB[7] could accelerate the dissolution rate by improving disintegration and β-CD showed a distinct advantage in solubility enhancement. Additionally, the drug release percentage of ZPF–CB[7] tablets was evidently greater in SIF (nearly 100%) than in SGF (<10% after 180 min). This finding suggested that the intestinal environment could trigger the release of prepared ZPF–CB[7] tablets. Furthermore, the effects of CB[7] on the dissolution of positively charged drugs should be evaluated systematically in the future because of the expected synergistic improvement in both solubility and disintegration.

## Discussion

In summary, the amorphous dispersions of ZPF co-ground with β-CD and CB[7] were successfully prepared through a ball grinding process to investigate their effects on the physicochemical properties of crystalline ZPF. Mono-ground ZPF was also prepared under the same grinding condition to eliminate the influence of polymorphism. Multiple characterization techniques, specifically UV-vis spectroscopy, molecular docking, XRPD, FI-IR, DSC, and ssNMR demonstrated the interactions between ZPF and macrocycles. The molecular arrangement of mono-ground ZPF was incompletely disordered and this arrangement slightly affected the solubility, disintegration, and dissolution rate of crystalline ZPF. Solubility and dissolution of crystalline ZPF can be significantly enhanced from co-ground dispersions (ZPF–CB[7] and ZPF–β-CD). Various blank tablets containing 0%–50% β-CD and CB[7] were prepared through direct powder compression to examine the effects of β-CD and CB[7] on oral tablets. Hardness tests revealed that the addition of β-CD and CB[7] increased the compressibility of the powder mixture, and the same weight of CB[7] elicited a stronger effect on compressibility than β-CD did. Disintegration tests demonstrated that more than 15% CB[7] effectively shortened the disintegrating time and acted as a disintegrant. *In vitro* dissolution tests showed that CB[7] accelerated the dissolution rate mainly by shortening the disintegrating time. β-CD displayed a distinct advantage in solubility enhancement. This study indicated that amorphous co-ground dispersions can effectively improve intrinsic drug solubility and dissolution. This study also revealed the enormous potential of CB[7] in the preparation of immediate-release tablets for future applications.

## Methods

### Materials

Glycoluril (98%) and paraformaldehyde powder (96%) were purchased from J&K Chemical (Beijing, China). β-CD (98%) and concentrated hydrogen chloride (HCl, 37%) were obtained from Chengdu Kelong Chemical Co., Ltd. (Chengdu, China). ZPF (98%) was purchased from Guangzhou Ciming Biological Technology Co., Ltd. (Guangzhou, China). All pharmaceutical excipients were kindly supplied by Sichuan Huirui Pharmaceutical Co., Ltd. (Chengdu, China). Tri-distilled water was used throughout the experiments.

### CB[7] synthesis

CB[7] was synthesized by a modified procedure of Bardelang *et al*.^36^ ^1^H NMR spectrum and MALDI-TOF-MS of synthetic CB[7] are shown in Figs S3 and S4, respectively. Elemental analysis for C_42_H_42_N_28_O_14_(H_2_O)_10_ is stated as follows: C 37.56, H 4.65, N 29.20%; found: C 37.61, H 4.64, N 29.09%.

### Recrystallization and grinding

Crystalline ZPF was obtained by recrystallization from anti-solvent system (dichloromethane/*n*-hexane). Mono-ground ZPF, co-ground dispersions ZPF–β-CD, and ZPF–CB[7] were prepared by grinding 1500 mg ZPF solely, 298 mg ZPF and 1135 mg β-CD, 298 mg ZPF and 1343 mg CB[7] respectively in a 50 mL agate jar with grinding balls (mixture of 10 × 10 mm, 20 × 5 mm; agate) for 5 h using a planetary mill (LNMN-QM 0.4L, Heishan Xinlitun Agate Handicrafts Co., Ltd., China) at room temperature. The rotation speed of the solar disk was set to 400 rpm, with alternate grinding periods (5 min) and pause periods (1 min). For comparison, corresponding binary physical mixtures with same ratio at 1:1 were prepared by simply mixing ZPF and β-CD or CB[7] for 5 min by using a vortex blender.

### UV-vis spectroscopy and molecular docking study

UV-vis spectroscopy was performed by using a TU-1901 UV-vis spectrophotometer (Persee, China) from 200–400 nm. Absorption spectra were recorded with various concentrations of β-CD (0, 0.5, 1.0, 1.5, 2.0, 3.0, 4.0, 6.0, 8.0 × 10^−3^ M) adding to a constant ZPF concentration (1 × 10^−4^ M). The interactions between β-CD and ZPF were determined by Benesi–Hildebrand equation:^37^


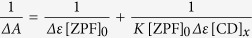


where Δ*A* is the change in absorbance of ZPF at 337 nm in the presence and absence of β-CD; Δ*ε* is the change in molar extinction coefficients between ZPF and its inclusion complex; [ZPF]_0_ and [CD]_x_ are the concentrations of ZPF and β-CD, respectively; and K is the binding constant that could be determined from the plot of 1/ΔA versus 1/[CD]_x_.

Theoretical studies are available to predict the favorable conformations of inclusion complexes and give insight into the interaction details. The structure of β-CD (CCDC No. 1107192) and CB[7] (CCDC No. 1428201) were abstracted from Cambridge Structural Database, and all attached water molecules were removed. The structure of ZPF was abstracted from the crystal structure that we solved. After which, molecular docking was conducted using Lamarckian genetic algorithm in AutoDock 4.0 program package (Accelrys). Docking simulations were performed with 200 runs, 2,500,000 energy evaluations, 27,000 numbers of generations, and 150 GA populations.

### Solid-state characterizations

XRPD analysis was performed at room temperature using an X’Pert PRO diffractometer (PANalytical Co., Ltd., Netherlands) with a PIXcel 1D detector and Cu K*α* radiation (λ = 1.54056 Å, generator setting: 40 kV and 40 mA). Diffraction data were collected at a step size of 0.01313° and a counting time of 30 ms/step.

FT-IR spectra were recorded on a FT-IR spectrometer (Nicolet 6700, Thermo Fisher Scientific) according to the KBr disc technique. Measurements were performed within the 4000 cm^−1^ to 400 cm^−1^ scanning range.

Differential scanning calorimetry (DSC) was conducted using a TA Q200 thermal analyzer (TA Instruments Co., New Castle, DE, USA). Samples in 5–8 mg were sealed in Al crucibles for DSC analysis at a 10 °C/min heating rate from 30 °C to 400 °C under nitrogen.

High resolution solid state ^13^C spectra of microcrystalline and mono-ground ZPF, CB[7], β-CD, and co-ground systems ZPF–CB[7] and ZPF–β-CD were recorded with a ramp cross polarization/magic angle spinning (CP-MAS) sequence. All ssNMR experiments were performed on a Bruker AV II-500 MHz NMR spectrometer operating at Larmor frequencies of 125.76 MHz for ^13^C and 500.13 MHz for ^1^H and equipped with a 4 mm (od) MAS probe at 303 K. ^13^C CP/MAS NMR chemical shifts were referenced with respect to tetramethylsilane at 0 ppm, using adamantine as secondary external standard. A spectral width of 37.9 kHz, recycle delay of 3 s, and acquisition time of 49.9 ms were used.

SEM was used to evaluate the morphological characteristics of the samples. Samples were coated with an electrically conducting thin layer of gold, then examined using a JSM-7500F SEM (JEOL, Japan) at 5.0 kV.

### Solubility studies

The effects of mono-ground ZPF, physical mixtures, and co-ground dispersions of β-CD and CB[7] on the solubility of crystalline ZPF were studied at 37.0 ± 0.1 °C in pure water, simulated gastric fluid (SGF; consisting of 2.0 g NaCl and 7.0 mL concentrated HCl per 1.0 L of water; pH = 1.1), and simulated intestinal fluid (SIF; consisting of 6.8 g of KH_2_PO_4_ and 0.89 g of NaOH in 1.0 L of water; pH = 6.8). An excess of crystalline and mono-ground ZPF, physical mixtures, and co-ground dispersions were added into water and buffered aqueous solutions. Suspensions were sonicated for 15 min then placed for 72 h in a water bath. After reaching equilibrium, the remaining solid was removed by filtration through a 0.45 μm membrane filter. The clear filtrates were suitably diluted and analyzed by UV–vis spectrophotometry at 337 nm. Each analysis was repeated three times.

### Blank tablets and ZPF tablets formulation

The formulation code and composition of blank tablets and ZPF tablets are shown in Table S2 and S3, respectively. The blank tablet formulation was designed referring to the report of Wheate^14^ as initial formulation to evaluate the effect of increasing content of β-CD and CB[7] on the tablets. Briefly, blank tablets containing 0%–50% lactose, corresponding 50%–0% β-CD or CB[7], 42.5% Avicel, 1% magnesium stearate, 2.5% talc, and 4% carboxyl methyl cellulose (CMC) were prepared by direct powder compression. ZPF tablets, containing 80 mg crystalline ZPF, mono-ground ZPF in the absence and presence of 20% (60 mg) β-CD or CB[7], and co-ground ZPF–β-CD/ZPF–CB[7] dispersions were prepared. When ZPF and macrocycles were added, lactose content was proportionally reduced, given that lactose is used as a diluent/bulking agent^38^. Before compression, powders were mixed thoroughly and all tablets at a weight of approximately 300 mg were prepared using a rotary tablet press (ZPS008, Shanghai Tianxiang & Chentai Pharmaceutical Machinery Co., Ltd., Shanghai, China) with a 9 mm diameter curved mold.

### Tablet hardness, disintegration and dissolution testing

Tablet hardness was measured using a conventional tablet hardness tester (0–20 kg, Shanghai Huanghai Drug Testing Instrument Co., Ltd.). Disintegrating time for each tablet was determined using a BJ–4 A intelligent disintegration apparatus (Tianjin Chuang Xing Electronic Equipment Manufacturing Co., Ltd.) by measuring the time for six tablets to disintegrate fully in 900 mL water, SGF, or SIF, at 37.0 ± 0.1 °C. Dissolution test was performed at 50 rpm and 37.0 ± 0.1 °C according to the paddle method, using 900 mL of water, SGF, and SIF as dissolution media. Aliquots (5 mL) from the sample solutions were obtained using a syringe at a prescribed time, filtered through a 0.45 μm hydrophilic membrane, and analyzed in a UV-vis spectrophotometer at 337 nm.

### Statistical analysis

Data were expressed as mean ± standard deviation. Statistical comparisons between the means of individual groups were performed through one-way ANOVA with Duncan’s test. Differences were considered statistically significant when ^*^p < 0.05. SPSS 11.0 software (SPSS, Chicago, IL, USA) was used for statistical analysis.

## Additional Information

**How to cite this article**: Li, S. *et al*. Co-grinding Effect on Crystalline Zaltoprofen with ß-cyclodextrin/Cucurbit[7]uril in Tablet Formulation. *Sci. Rep.* **7**, 45984; doi: 10.1038/srep45984 (2017).

**Publisher's note:** Springer Nature remains neutral with regard to jurisdictional claims in published maps and institutional affiliations.

## Supplementary Material

Supporting Information

## Figures and Tables

**Figure 1:**
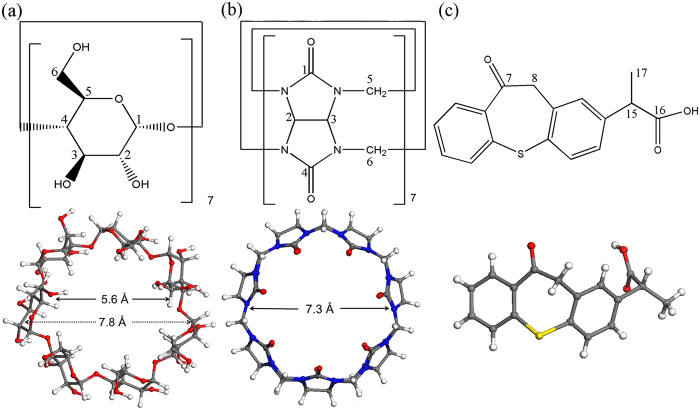
The chemical structure of (**a**) β-CD, (**b**) CB[7] and (**c**) ZPF.

**Table 1:** Solubility of microcrystalline ZPF, mono-ground ZPF and the corresponding physical mixtures (ZPF + CB[7] and ZPF + β-CD) and co-ground dispersions (ZPF−CB[7], ZPF−β-CD) in pure water, SGF and SIF at 37 ± 0.1 °C after 72 h.

Samples	Solubility (mg/mL)		
	Water	SGF	SIF
Microcrystalline ZPF	0.018 ± 0.003	0.003 ± 0.001	2.23 ± 0.132
Mono-ground ZPF	0.021 ± 0.005	0.005 ± 0.001	2.37 ± 0.051
Microcrystalline ZPF + CB[7]	0.023 ± 0.004	0.008 ± 0.002	1.19 ± 0.035^&^
Microcrystalline ZPF + β-CD	0.090 ± 0.008^*^	0.022 ± 0.004^#^	1.45 ± 0.041^&^
Mono-ground ZPF + CB[7]	0.025 ± 0.005	0.009 ± 0.003	1.28 ± 0.079^&^
Mono-ground ZPF + β-CD	0.093 ± 0.007^*^	0.022 ± 0.006^#^	1.49 ± 0.086^&^
ZPF−CB[7]	0.076 ± 0.006^*^	0.052 ± 0.005^#^	1.23 ± 0.082^&^
ZPF−β-CD	0.101 ± 0.013^*^	0.063 ± 0.006^#^	1.58 ± 0.051^&^

Values are presented as mean ± SD (n = 3), ^*^p < 0.05 compared with microcrystalline ZPF group in water; ^#^*p* < 0.05 com*p*ared with microcrystalline ZPF group in SGF; ^&^*p* < 0.05 compared with microcrystalline ZPF group in SIF.

**Table 2:** The tablet thickness and disintegrating time of blank tablets containing 0%, 5%, 15%, 25% and 50% CB[7] or β-CD in pure water.

	F0	β-CD				CB[7]			
	0%	5%	15%	25%	50%	5%	15%	25%	50%
Tablet thickness (mm)	2.6	2.6	2.8	3.0	3.1	2.8	3.0	3.2	3.7
Disintegrating time (min)	36	34	33	30	32	32	5	2	<1

**Table 3:** The disintegrating time of various ZPF tablets (each tablet containing 20% CB[7] or β-CD, and 80 mg ZPF) in water, SGF and SIF.

Samples	Disintegrating time (min)		
	Water	SGF	SIF
Microcrystalline ZPF tablet	31	1	27
Mono-ground ZPF tablet	26	1	21
Microcrystalline ZPF + CB[7] tablet	<1	<1	<1
Microcrystalline ZPF + β-CD tablet	33	<1	36
Mono-ground ZPF + CB[7] tablet	<1	<1	<1
Mono-ground ZPF + β-CD tablet	29	<1	31
ZPF−CB[7] tablet	<1	<1	<1
ZPF−β-CD tablet	30	<1	33
