# Supplementary material for: Co-grinding Effect on Crystalline Zaltoprofen with β-cyclodextrin/Cucurbit[7]uril in Tablet Formulation
Source: Sci Rep. 2017 Apr 3;7:45984. doi: 10.1038/srep45984 (PMC5377470; doi:10.1038/srep45984)

Supporting Information for

# Co-grinding Effect on Crystalline Zaltoprofen with β-cyclodextrin/Cucurbit[7]uril in Tablet Formulation

Shanshan Li, Xiang Lin, Kailin Xu, Jiawei He, Hongqin Yang, Hui Li*

## 1. CB[7] preparation

10 g glycoluril was stirred with 14.2 mL 37% HCl solution in a 50 mL round-bottomed flask. Then finely powdered paraformaldehyde (4.22 g) was slowly added. After 30 min vigorous stirring, the viscous solution set as a gel, which then was refluxed for 18 h at 100 °C. The reaction mixture was cooled to room temperature, and a large amount of precipitate formed. After filtration, ~ 40 mL water was added to the filtrate and allowed insoluble CB[*n*]s and impurities to precipitate. Then the CB[7] dissolved filtrate was reduced to 10 mL volume and 80 mL methanol was added causing immediate formation of a white precipitate (Crude CB[7]). The precipitate was filtered and suspended in 200 mL of 20% aqueous glycerol and the solution was heated to 80 °C under stirring for 3 h. The mixture was then filtered and 200 mL methanol was added into the colorless solution and stirred overnight. The white precipitate was formed and suction filtered using 0.45 μm membrane with plenty of methanol. Finally, the solid (CB[7]) was dried under 100 °C for 24 h to remove the methanol (Yield: 1.5 g).

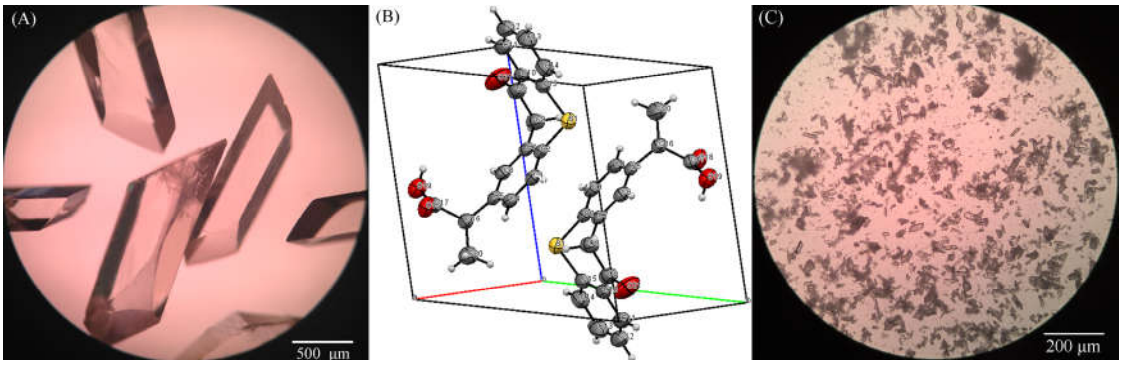


**Figure S1. (A) Crystalline ZPF for single-crystal X-ray diffraction; (B) crystal structure of the crystalline ZPF; and (C) microcrystalline ZPF for tablet formulation.**

**Table S1. Crystallographic data for crystalline ZPF.**

| Empirical Formula | $C_{17}H_{14}O_3S$ |
|---|---|
| Formula weight | 298.34 |
| Crystal system | Triclinic |
| Space group | *P*-1 |
| $a$/Å | 8.4369(6) |
| $b$/Å | 9.4067(9) |
| $c$/Å | 10.1570(5) |
| $\alpha$/° | 109.951(7) |
| $\beta$/° | 102.505(6) |
| $\gamma$/° | 98.345(7) |
| Volume/Å$^3$ | 718.36(9) |
| $Z$ | 2 |
| $D$c/g.cm$^{-3}$ | 1.379 |
| $F$(000) | 312.0 |
| μ/mm$^{-1}$ | 0.232 |
| Crystal size/mm$^3$ | 0.40 × 0.40 × 0.35 |
| GOF | 1.053 |
| $R_1$ [$I \geq 2\sigma(I)$] | 0.0517 |
| w$R_2$ [all data] | 0.1475 |

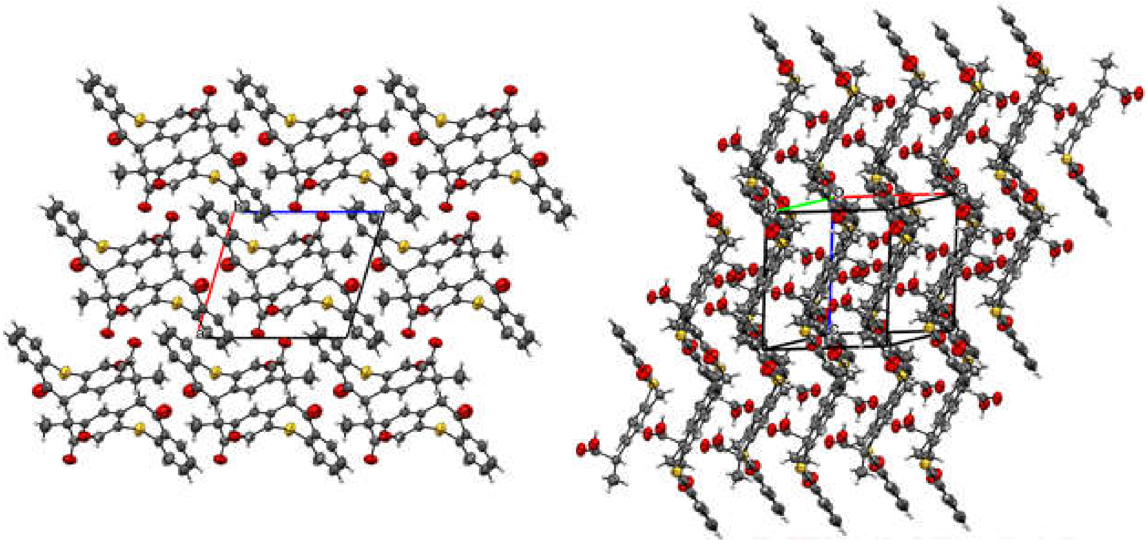

**Figure S2. Perspective views of 3D packing structures of crystalline ZPF in different directions.**

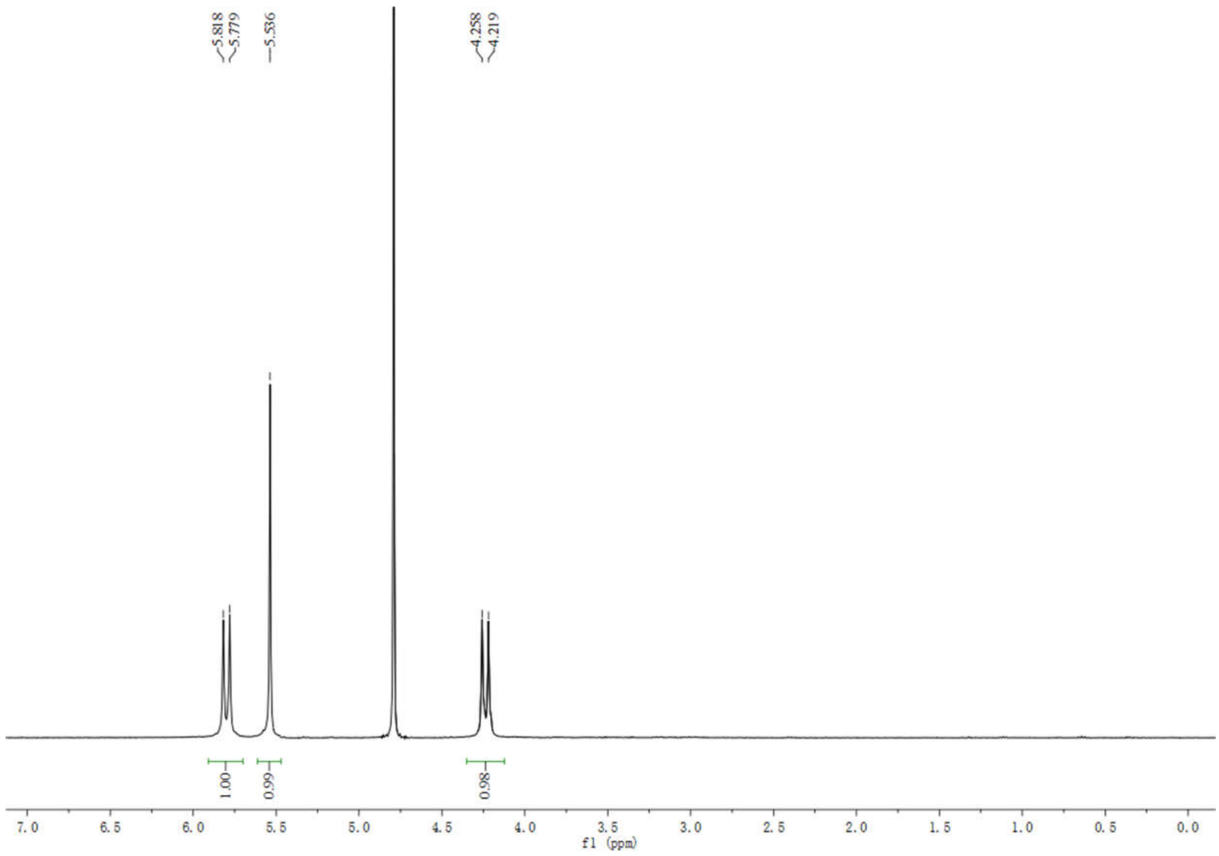


**Figure S3. $^1$H NMR spectrum of the synthetic CB[7] in $D_2O$.**

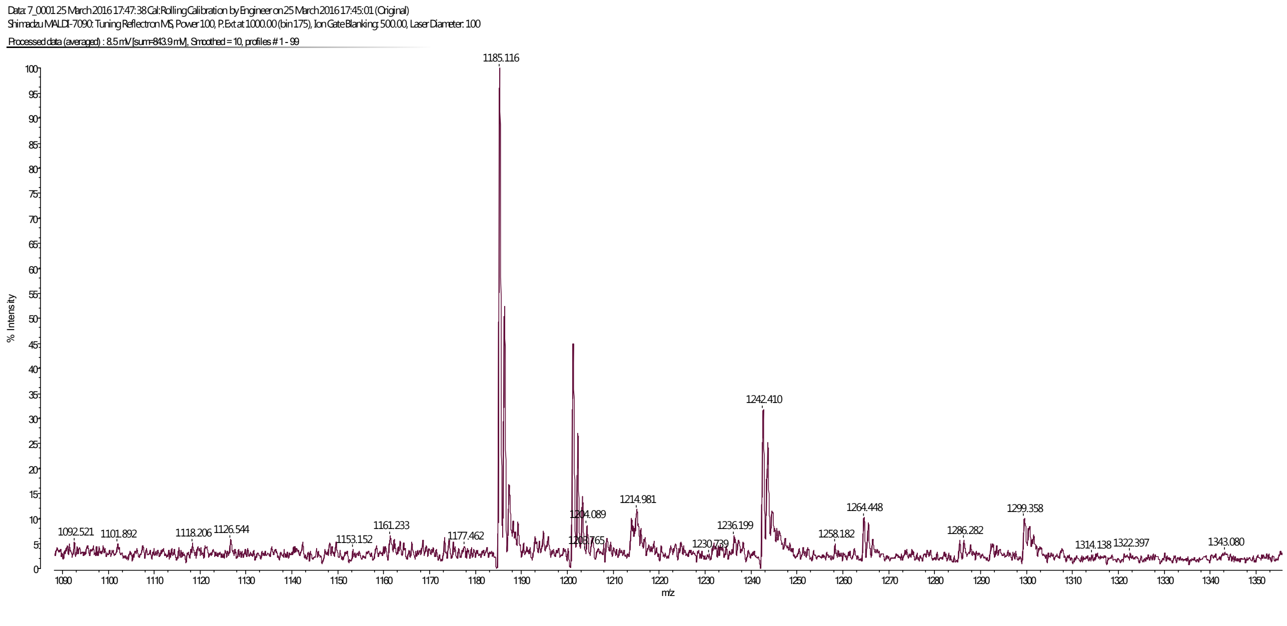


**Figure S4. MALDI-TOF-MS of the synthetic CB[7].**

**Table S2. Composition of blank tablets with different contents of β-CD or CB[7].**

| Composition | Fomulation code | | | | |
|---|---|---|---|---|---|
| | F0 | F1* | F2* | F3* | F4* |
| Lactose | 50% | 45% | 35% | 25% | – |
| β-CD/CB[7] | – | 5% | 15% | 25% | 50% |
| Avicel | 42.5% | 42.5% | 42.5% | 42.5% | 42.5% |
| Magnesium stearate | 1% | 1% | 1% | 1% | 1% |
| Talc | 2.5% | 2.5% | 2.5% | 2.5% | 2.5% |
| CMC | 4% | 4% | 4% | 4% | 4% |

* F*n* (*n* = 1−4) contains $F_D n$ and $F_B n$ for β-CD blank tablets and CB[7] blank tablets, respectively.

**Table S3. Composition of various ZPF tablets.**

| Composition | Fomulation code (mg) | | | | | | | |
|---|---|---|---|---|---|---|---|---|
| | F5* | F6* | F7* | F8* | F9* | F10* | F11* | F12* |
| ZPF | $80_{(C)}$# | $80_{(M)}$# | $80_{(C)}$ | $80_{(C)}$ | $80_{(M)}$ | $80_{(M)}$ | 140 | 140 |
| β-CD/CB[7] | – | – | $60_{β\text{-CD}}$ | $60_{CB[7]}$ | $60_{β\text{-CD}}$ | $60_{CB[7]}$ | ZPF−β-CD dispersion | ZPF−CB[7] dispersion |
| Lactose | 70 | 70 | 10 | 10 | 10 | 10 | 10 | 10 |
| Avicel | 127.5 | 127.5 | 127.5 | 127.5 | 127.5 | 127.5 | 127.5 | 127.5 |
| Magnesium stearate | 3 | 3 | 3 | 3 | 3 | 3 | 3 | 3 |
| Talc | 7.5 | 7.5 | 7.5 | 7.5 | 7.5 | 7.5 | 7.5 | 7.5 |
| CMC | 12 | 12 | 12 | 12 | 12 | 12 | 12 | 12 |

* F*n* (*n* = 5−12) represent crystalline ZPF tablet, mono-ground ZPF tablet, crystalline ZPF + β-CD tablet, crystalline ZPF + CB[7] tablet, mono-ground ZPF + β-CD tablet, mono-ground ZPF + CB[7] tablet, co-ground ZPF−β-CD tablet and co-ground ZPF−CB[7] tablet, respectively.

# (C) means the crystalline ZPF and (M) means the mono-ground ZPF.

# checkCIF/PLATON report

You have not supplied any structure factors. As a result the full set of tests cannot be run.

THIS REPORT IS FOR GUIDANCE ONLY. IF USED AS PART OF A REVIEW PROCEDURE FOR PUBLICATION, IT SHOULD NOT REPLACE THE EXPERTISE OF AN EXPERIENCED CRYSTALLOGRAPHIC REFEREE.

No syntax errors found. CIF dictionary Interpreting this report

# Datablock: I

---

```
Bond precision:  C-C = 0.0045 A          Wavelength=0.71073

Cell:          a=8.4504(9)         b=9.4152(11)      c=10.1820(13)
               alpha=110.015(11)  beta=102.496(11)  gamma=98.224(10)
Temperature:  293 K

                Calculated                 Reported
Volume          722.03(17)                 722.03(15)
Space group     P -1                       P -1
Hall group      -P 1                       -P 1
Moiety formula  C17 H14 O3 S               C17 H14 O3 S
Sum formula     C17 H14 O3 S               C17 H14 O3 S
Mr              298.34                     298.34
Dx,g cm-3       1.372                      1.372
Z               2                          2
Mu (mm-1)       0.231                      0.231
F000            312.0                      312.0
F000'           312.39
h,k,lmax        10,11,12                   10,11,12
Nref            2959                       2950
Tmin,Tmax       0.946,0.955                0.867,1.000
Tmin'           0.933

Correction method= # Reported T Limits: Tmin=0.867 Tmax=1.000
AbsCorr = MULTI-SCAN

Data completeness= 0.997          Theta(max)= 26.370

R(reflections)= 0.0574( 2006)     wR2(reflections)= 0.1689( 2950)

S = 1.062                  Npar= 191
```

---

The following ALERTS were generated. Each ALERT has the format
**test-name_ALERT_alert-type_alert-level**.
Click on the hyperlinks for more details of the test.

### Alert level B

```
PLAT415_ALERT_2_B Short Inter D-H..H-X       H13    ..  H19     ..       2.01 Ang.
PLAT420_ALERT_2_B D-H Without Acceptor       O19    --  H19    ...     Please Check
```

### Alert level C

```
CRYSC01_ALERT_1_C The word below has not been recognised as a standard
            identifier.
            transparent
CRYSC01_ALERT_1_C No recognised colour has been given for crystal colour.
PLAT230_ALERT_2_C Hirshfeld Test Diff for    C10    --  C15     ..        5.5 s.u.
PLAT230_ALERT_2_C Hirshfeld Test Diff for    C12    --  C13     ..        6.7 s.u.
PLAT340_ALERT_3_C Low Bond Precision on  C-C Bonds ...............     0.0045 Ang.
```

### Alert level G

```
PLAT005_ALERT_5_G No Embedded Refinement Details found  in the CIF     Please Do !
PLAT007_ALERT_5_G Number of Unrefined Donor-H Atoms ..............          1 Report
PLAT152_ALERT_1_G The Supplied and Calc. Volume s.u. Differ by ...          2 Units
PLAT199_ALERT_1_G Reported _cell_measurement_temperature ..... (K)        293 Check
PLAT200_ALERT_1_G Reported   _diffrn_ambient_temperature ..... (K)        293 Check
PLAT793_ALERT_4_G The Model has Chirality at C16     (Centro SPGR)          S Verify
```

0 **ALERT level A** = Most likely a serious problem - resolve or explain
2 **ALERT level B** = A potentially serious problem, consider carefully
5 **ALERT level C** = Check. Ensure it is not caused by an omission or oversight
6 **ALERT level G** = General information/check it is not something unexpected

5 ALERT type 1 CIF construction/syntax error, inconsistent or missing data
4 ALERT type 2 Indicator that the structure model may be wrong or deficient
1 ALERT type 3 Indicator that the structure quality may be low
1 ALERT type 4 Improvement, methodology, query or suggestion
2 ALERT type 5 Informative message, check

## checkCIF publication errors

### Alert level A

```
PUBL002_ALERT_1_A  The contact author’s address is missing,
            _publ_contact_author_address.
PUBL005_ALERT_1_A  _publ_contact_author_email, _publ_contact_author_fax and
            _publ_contact_author_phone are all missing.
            At least one of these should be present.
PUBL006_ALERT_1_A  _publ_requested_journal is missing
            e.g. ’Acta Crystallographica Section C’
PUBL008_ALERT_1_A  _publ_section_title is missing. Title of paper.
PUBL009_ALERT_1_A  _publ_author_name is missing. List of author(s) name(s).
PUBL010_ALERT_1_A  _publ_author_address is missing. Author(s) address(es).
PUBL012_ALERT_1_A  _publ_section_abstract is missing.
            Abstract of paper in English.
```

7 **ALERT level A** = Data missing that is essential or data in wrong format
0 **ALERT level G** = General alerts. Data that may be required is missing

**Publication of your CIF**

You should attempt to resolve as many as possible of the alerts in all categories. Often the minor alerts point to easily fixed oversights, errors and omissions in your CIF or refinement strategy, so attention to these fine details can be worthwhile. In order to resolve some of the more serious problems it may be necessary to carry out additional measurements or structure refinements. However, the nature of your study may justify the reported deviations from journal submission requirements and the more serious of these should be commented upon in the discussion or experimental section of a paper or in the "special_details" fields of the CIF. *checkCIF* was carefully designed to identify outliers and unusual parameters, but every test has its limitations and alerts that are not important in a particular case may appear. Conversely, the absence of alerts does not guarantee there are no aspects of the results needing attention. It is up to the individual to critically assess their own results and, if necessary, seek expert advice.

If level A alerts remain, which you believe to be justified deviations, and you intend to submit this CIF for publication in a journal, you should additionally insert an explanation in your CIF using the Validation Reply Form (VRF) below. This will allow your explanation to be considered as part of the review process.

**Validation response form**

Please find below a validation response form (VRF) that can be filled in and pasted into your CIF.

```
# start Validation Reply Form
_vrf_PUBL002_GLOBAL
;
PROBLEM: The contact author’s address is missing,
RESPONSE: ...
;
_vrf_PUBL005_GLOBAL
;
PROBLEM: _publ_contact_author_email, _publ_contact_author_fax and
RESPONSE: ...
;
_vrf_PUBL006_GLOBAL
;
PROBLEM: _publ_requested_journal is missing
RESPONSE: ...
;
_vrf_PUBL008_GLOBAL
;
PROBLEM: _publ_section_title is missing. Title of paper.
RESPONSE: ...
;
_vrf_PUBL009_GLOBAL
;
PROBLEM: _publ_author_name is missing. List of author(s) name(s).
RESPONSE: ...
;
_vrf_PUBL010_GLOBAL
;
PROBLEM: _publ_author_address is missing. Author(s) address(es).
RESPONSE: ...
;
_vrf_PUBL012_GLOBAL
;
```

```
PROBLEM: _publ_section_abstract is missing.
RESPONSE: ...
;
# end Validation Reply Form
```

If you wish to submit your CIF for publication in Acta Crystallographica Section C or E, you should upload your CIF via the web. If you wish to submit your CIF for publication in IUCrData you should upload your CIF via the web. If your CIF is to form part of a submission to another IUCr journal, you will be asked, either during electronic submission or by the Co-editor handling your paper, to upload your CIF via our web site.

---

## PLATON version of 24/11/2016; check.def file version of 23/11/2016

**Datablock I** - ellipsoid plot

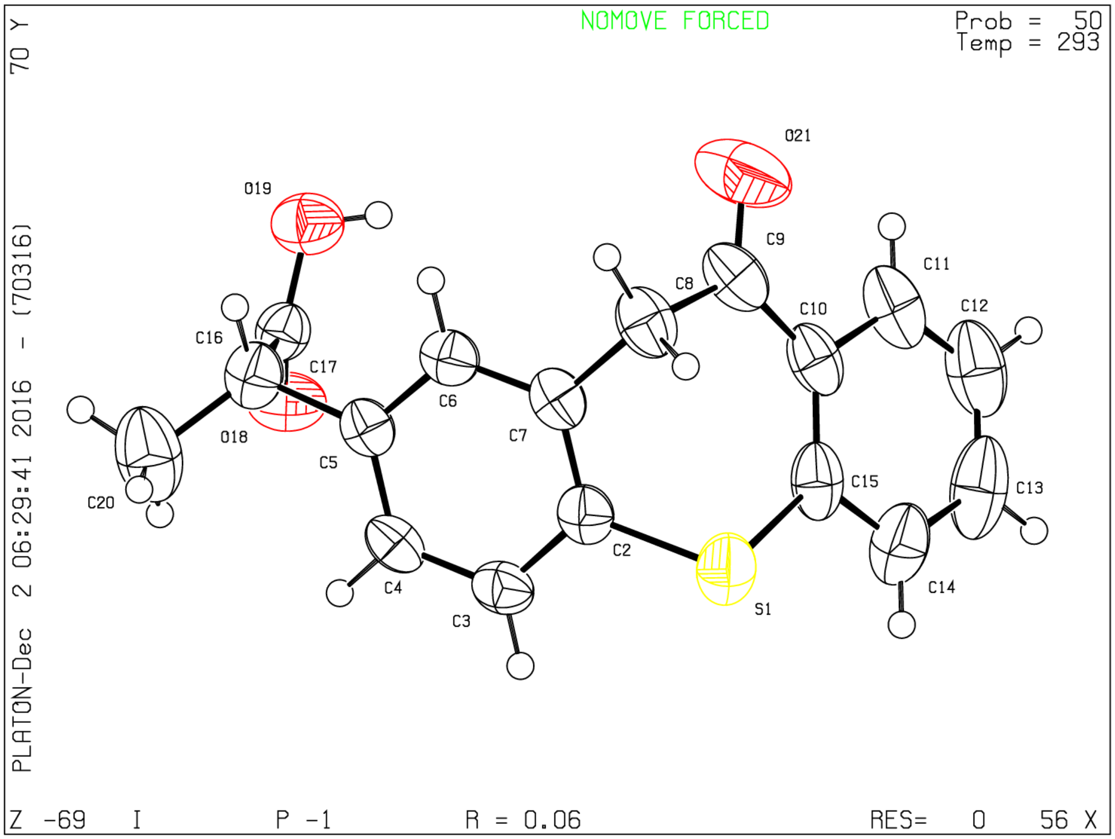

Supplement: Supporting Information [file srep45984-s1.pdf]
